# SARS-CoV-2: The Interplay Between Evolution and Host Immunity

**DOI:** 10.1146/annurev-immunol-083122-043054

**Published:** 2024-12-20

**Authors:** James Brett Case, Shilpi Jain, Mehul S. Suthar, Michael S. Diamond

**Affiliations:** 1 Department of Medicine, Washington University School of Medicine, St. Louis, Missouri, USA; 2 Center for Childhood Infections and Vaccines of Children’s Healthcare of Atlanta, Department of Pediatrics, Emory University School of Medicine, Atlanta, Georgia, USA; 3 Emory Vaccine Center, Emory National Primate Research Center, Atlanta, Georgia, USA; 4 Department of Pathology & Immunology; Department of Molecular Microbiology; and Andrew M. and Jane M. Bursky Center for Human Immunology and Immunotherapy Programs, Washington University School of Medicine, St. Louis, Missouri, USA

**Keywords:** SARS-CoV-2, evolution, immunity, protection, vaccines, variants

## Abstract

The persistence of SARS-CoV-2 infections at a global level reflects the repeated emergence of variant strains encoding unique constellations of mutations. These variants have been generated principally because of a dynamic host immune landscape, the countermeasures deployed to combat disease, and selection for enhanced infection of the upper airway and respiratory transmission. The resulting viral diversity creates a challenge for vaccination efforts to maintain efficacy, especially regarding humoral aspects of protection. Here, we review our understanding of how SARS-CoV-2 has evolved during the pandemic, the immune mechanisms that confer protection, and the impact viral evolution has had on transmissibility and adaptive immunity elicited by natural infection and/or vaccination. Evidence suggests that SARS-CoV-2 evolution initially selected variants with increased transmissibility but currently is driven by immune escape. The virus likely will continue to drift to maintain fitness until countermeasures capable of disrupting transmission cycles become widely available.

## INTRODUCTION

The emergence of SARS-CoV-2 in late 2019 resulted in a global pandemic, an enormous loss of life, economic hardship, and social disruption. SARS-CoV-2, which causes COVID-19, is transmitted through respiratory droplets and infects epithelial cells within the respiratory tract (1–5). Acute infection with SARS-CoV-2 can result in an array of clinical syndromes, ranging from asymptomatic infection to rapidly progressive respiratory and/or multi-organ failure resulting in death. It is well documented that disease severity and infection outcome are dictated by the intensity and quality of the innate and adaptive immune responses, which may be influenced by age, comorbidities, and preexisting immunity (3).

SARS-CoV-2 is a betacoronavirus; this genus in the *Coronaviridae* family includes other highly lethal human coronaviruses (CoVs), such as SARS-CoV and MERS-CoV, but also viruses such as HKU1 and HCoV-OC43, which cause seasonal and often mild human respiratory tract infections (6). SARS-CoV-2 cell entry is mediated by the spike attachment glycoprotein, which is displayed on the virion surface. Upon engagement with its dominant host cell receptor, angiotensin-converting enzyme 2 (ACE2), which is expressed on ciliated epithelial cells or type II pneumocytes in the respiratory tract, the spike protein undergoes conformational changes resulting in membrane fusion and viral entry. As the spike protein is the immunodominant antigen, multiple vaccines and passive antibody-based therapeutics that target it have been developed and deployed. Nonetheless, SARS-CoV-2 evolution, which is driven by multiple factors, has complicated global efforts to reduce viral transmission and prevent disease.

CoVs, like many RNA viruses, can evolve in response to changes in their environment, including host immunity, antiviral interventions, and adaptation to new host species. RNA virus evolution can occur through the introduction of point mutations, insertions, deletions, or recombination events and enable the emergence of new viral strains or variants, some with unique or advantageous genetic traits. Many RNA viruses exhibit high genome mutation rates (7), a characteristic attributed to their error-prone viral RNA-dependent RNA polymerases (RdRps) and the absence of proofreading enzymes such as those encoded by DNA-based organisms (7–9). As a result, individual RNA virus genomes accumulate mutations on the order of 10^−3^ to 10^−5^ mutations per nucleotide per replication cycle, resulting in populations of genetically similar RNA viruses, commonly referred to as quasispecies or mutant swarms. While the rapid generation of sequence diversity may benefit RNA viruses by allowing them to overcome environmental and immune challenges, there is a balance between too much and too little diversity. Too much diversity occurring too rapidly can lead to error catastrophe, also known as lethal mutagenesis, wherein deleterious mutations affect key steps in the viral life cycle. Therefore, RNA viruses likely exist within an optimal window of sequence space that balances genetic diversity with genome stability and yet enables adaptation to new species, immune system pressures, and host-to-host transmission cycles (8). In this review, we highlight the mechanisms used by SARS-CoV-2 to generate viral diversity, the factors that drive adaptation, and the effects of viral evolution on host immunity.

### SARS-CoV-2 Variant Evolution

**Figure 1:**
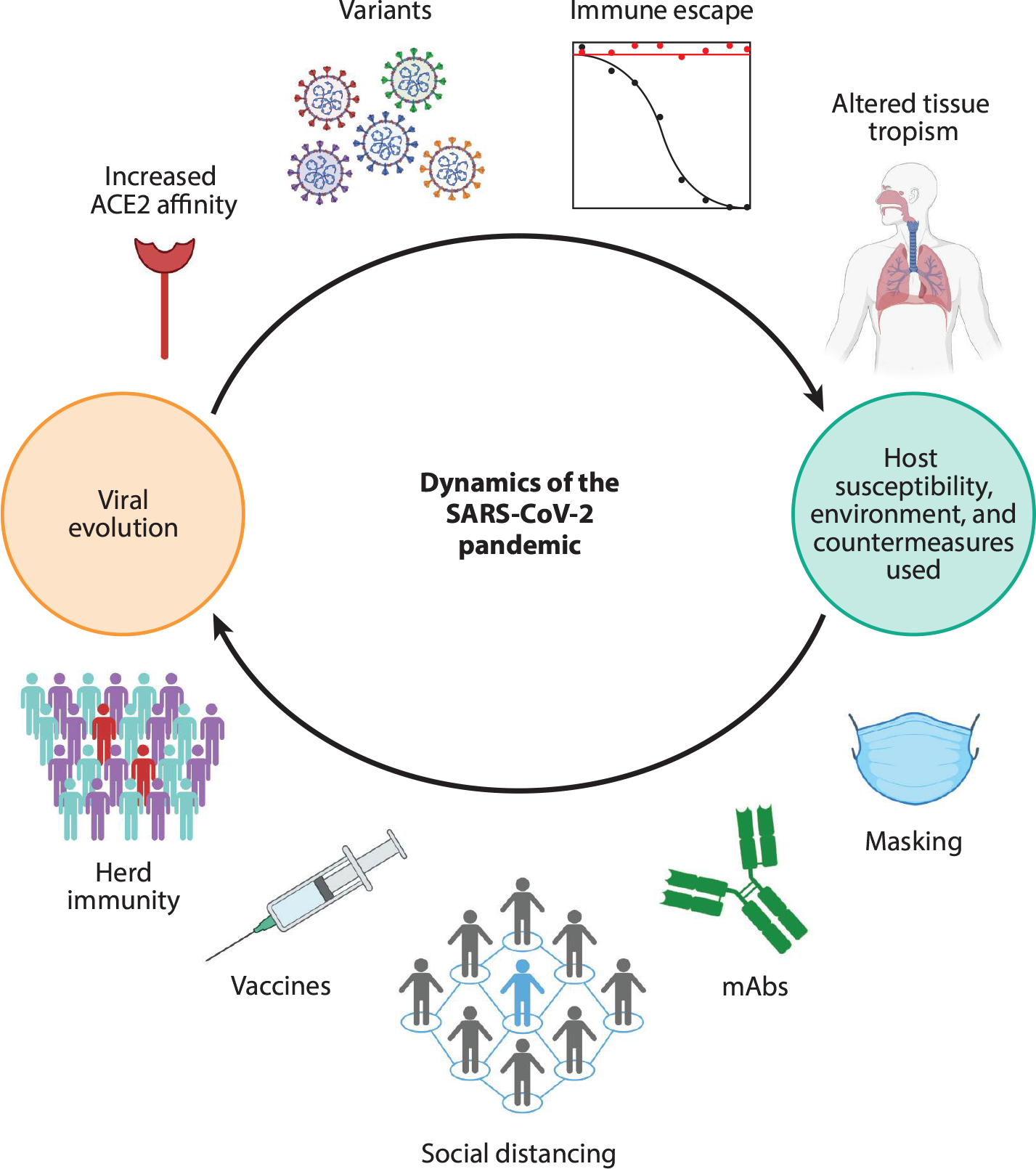
Interdependence of viral evolution and behavioral, biological, and immunological pressures. Environmental pressures, including behavioral activities (masking and social distancing), deployed countermeasures (mAbs, vaccines, and others), and preexisting immunity can promote virus adaptations. SARS-CoV-2 has evolved unique constellations of mutations for increased transmissibility and immune escape, largely against humoral immunity. Abbreviations: ACE2, angiotensin-converting enzyme 2; mAbs, monoclonal antibodies. Figure adapted from images created with BioRender.com.

**Figure 2:**
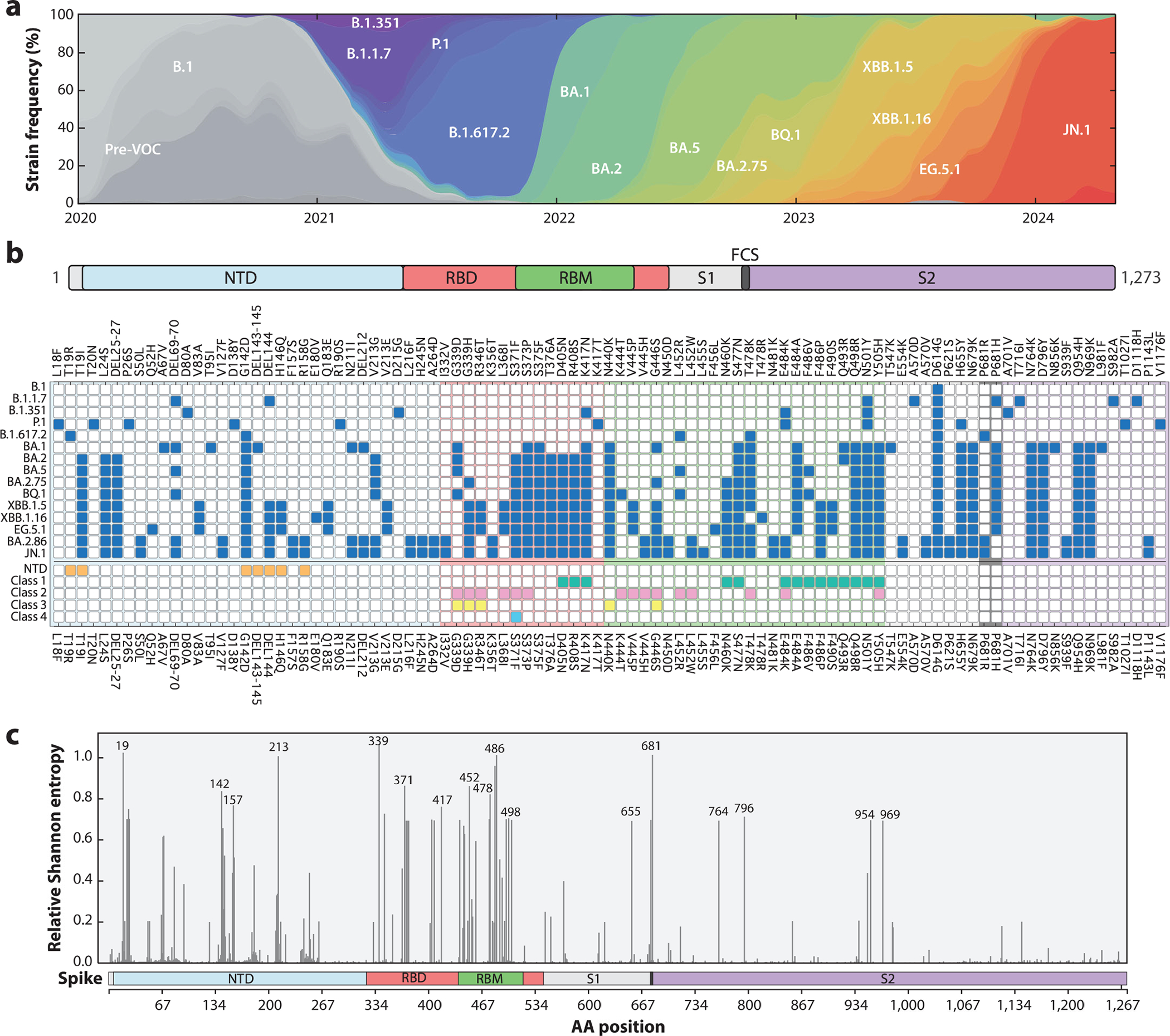
SARS-CoV-2 variant emergence and mutational identity. (*a*) Following the emergence of the SARS-CoV-2 Wuhan-1 strain in late 2019, antigenic drift, recombination, and potentially other evolutionary mechanisms have resulted in repeated waves of dominant variants. (*b*) A schematic representation of the SARS-CoV-2 spike protein with relevant regions labeled. The AA substitutions present within the selected strains are indicated, including mutations associated with antibody escape. (*c*) Shannon entropy plot indicating the mutational heterogeneity at each individual AA residue. Residues with notably high levels of entropy are labeled. Abbreviations: AA, amino acid; FCS, furin cleavage site; NTD, N-terminal domain; RBD, receptor-binding domain; RBM, receptor-binding motif; S1, spike domain 1; S2, spike domain 2; VOC, variant of concern. Panels *a* and *c* generated using sequences submitted to GISAID.org and visualized using Nextstrain.org (CC BY 4.0) (24).

Global surveillance and sequencing efforts have provided a wealth of genetic information on SARS-CoV-2 evolution. During the first several months of the COVID-19 pandemic, the rate of viral evolution, in the absence of immune pressures, was relatively low (2 × 10^−6^ per site per day) (10–12). However, social behaviors (the introduction of masking and social distancing), host immune status (naive populations, vaccinated individuals, and herd immunity), the development of passive countermeasures to treat vulnerable immunocompromised hosts, and the resulting selective pressures shaped the evolutionary trajectory of the virus (Figure 1). By March 2020, a strain encoding a D614G mutation with increased transmission capacity had supplanted the original strain (Figure 2a) (13). In late 2020 to early 2021, the Alpha, Beta, Gamma, and Delta variants independently emerged from different regions of the world, each encoding a set of unique mutations that conferred increased transmissibility and/or immune escape properties (Figure 2a,b) (14–17). In particular, the SARS-CoV-2 spike protein has exhibited extensive heterogeneity at many positions, which can be quantified by the relative Shannon entropy (Figure 2c), a statistical measure of the sequence variation present at individual residues (18). Beginning in 2021, a sharp increase (3.6% of residues to 8.1% of residues) in diversifying pressure was seen in the receptor-binding domain (RBD) (19). In November 2021, the Omicron variant, which initially was composed of five sublineages (BA.1, BA.2, BA.3, BA.4, and BA.5), emerged in southern Africa (20). In contrast to previous variants, which encoded roughly 10 to 15 nonsynonymous spike mutations relative to the ancestral SARS-CoV-2 sequence, first-generation Omicron variant lineages encoded more than 30, many of which were present at residues that facilitate antibody escape (Figure 2b) (20). Throughout 2022, variant BA.2, variant BA.5, and their descendants (BA.2.75 and BQ.1.1, respectively) cycled as globally dominant strains (21). Since early 2023, after the emergence of the XBB variants, all successive waves of dominant variants have been descendants of the BA.2 lineage. As of June 2024, the Omicron lineage has been dominant for more than half of the pandemic, and current evolutionary patterns suggest that gradual antigenic drift will continue, as evidenced by a stepwise accumulation of mutations in distinct, but related, Omicron variants over the last 2 years (22, 23). At the time of this writing, BA.2-lineage descendant variants KP.2, KP.3, and XEC are emerging and replacing JN.1 variants.

### Factors that Drive SARS-CoV-2 Evolution

While the source of variant evolution with extensive immune escape properties remains unclear, chronic infections in immunocompromised hosts, spillover-spillback events between humans and animal reservoirs, and/or undetected circulation in regions with low surveillance levels may be the origin(s) (12). SARS-CoV-2 exhibits a broad host range, which beyond humans includes domesticated animals (e.g., dogs, cats, ferrets, and hamsters) and wildlife (e.g., white-tailed deer, mink, lions, and tigers) (25). In 2021, outbreaks in several mink farms were reported in Europe and the United States, resulting in mass culling to prevent further spread (26–29). Analysis of SARS-CoV-2 genomes from mink suggests that some mutations (e.g., spike Y453F) are adaptive, conferring increased human ACE2 (hACE2) receptor binding and evasion against convalescent SARS-CoV-2 human sera (30, 31). These data indicate that human-to-mink zoonoses and/or spillover-spillback events into humans are possible and could be a potential source of viral diversity.

## ADAPTIVE IMMUNE PROTECTION AGAINST SARS-COV-2 INFECTION AND DISEASE

An understanding of the factors that drive SARS-CoV-2 evolution requires an exploration of the immune mechanisms of control, which are a dominant source of selective pressure. Broad, durable, and potent B cell and T cell responses as well as immunological memory are necessary for protection against reinfection by COVID-19. It is estimated that more than 96% of the adult population in the United States has acquired immunity derived from infection and/or vaccination against SARS-CoV-2 (32). While immunity conferred by natural infection or vaccination alone shows variable efficacy against subsequent variant exposure, several studies have reported that hybrid immunity (a combination of immunization and natural infection-induced immunity) provides more durable protection against hospitalization and severe disease (33–35). In this section, we highlight the aspects of protective adaptive immunity elicited from infection and vaccination, which preferentially serve as the selective pressures for evolution of variant SARS-CoV-2 strains. Note that innate immune responses also contribute to protection against SARS-CoV-2 infection but have been reviewed in detail elsewhere (36, 37).

### Humoral and B Cell Immunity Against SARS-CoV-2

**Figure 3:**
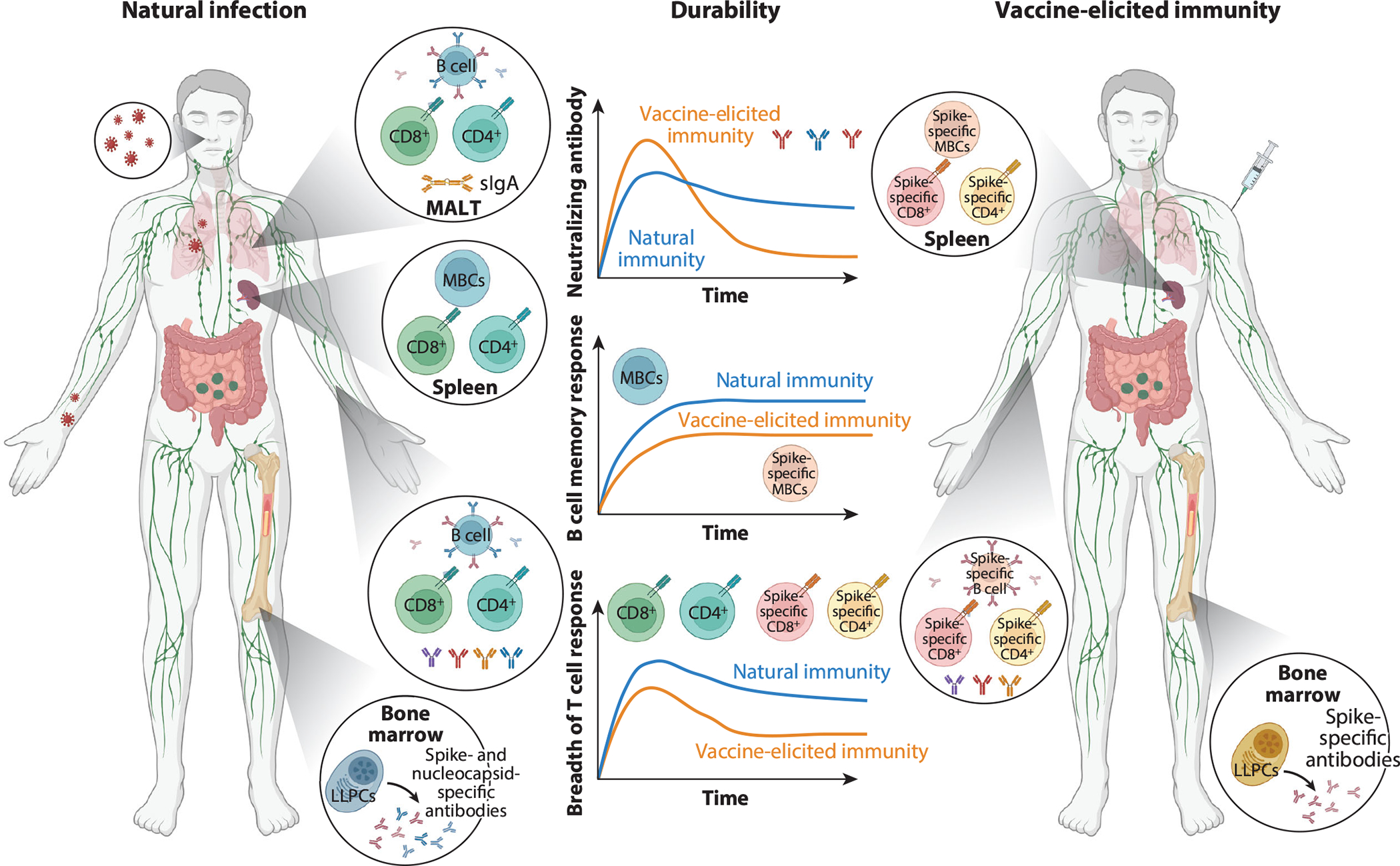
Adaptive immunity against SARS-CoV-2. Following infection with SARS-CoV-2, antigen-specific B and T cells are induced systemically and in mucosal tissues. Plasmablasts and LLPCs produce neutralizing antibodies, which prevent reinfection with homologous or closely related strains for months. MBCs and memory T cells are maintained in multiple sites throughout the body and rapidly respond to cognate or variant antigen following reinfection. Mucosal antigen exposure through natural infection elicits sIgA production within respiratory tissues. Systemic vaccination, via intramuscular injection, elicits higher levels of neutralizing antibodies in serum with more rapid rates of decay relative to natural infection. Mucosal immunity is notably absent following intramuscular vaccination. Abbreviations: LLPCs, long-lived plasma cells; MALT, mucosa-associated lymphoid tissue; MBCs, memory B cells; sIgA, secretory IgA. Figure adapted from images created with BioRender.com.

SARS-CoV-2 infection leads to rapid generation of inhibitory systemic and mucosal antibody responses. SARS-CoV-2 spike-specific antibodies appear in the blood within days of symptom onset and subsequently peak around 21 days later (Figure 3) (38–40). The presence of RBD-binding antibodies in serum correlates with virus neutralization. The magnitude of spike-specific antibodies associates with disease severity, likely as a reflection of viral antigen burden, although preexisting conditions (e.g., diabetes, obesity, cancer, and use of immunosuppressive therapeutics) and age can influence the magnitude of the antibody response (41–43).

Longitudinal and cross-sectional adult cohort studies have reported that circulating spike-specific IgG antibodies and neutralizing antibodies persist in the blood for months after infection, serving as a selective pressure upon reencounter with a closely or distantly related variant. The decay rates of spike-specific IgG and neutralizing antibodies show a half-life ranging from 140 to 364 days, whereas the half-life of IgA spike-specific antibodies is about 300 days (39, 44–48). Age and comorbidities, such as cancer, affect the magnitude of the half-life of IgG-specific and neutralizing antibodies (49–56), which can result in subpopulations that have different (more or less) immune pressures on the virus.

Mucosa-associated lymphoid tissue is a component of the immune system in the upper respiratory tract. Here, multimeric IgA antibodies are generated and transported across the mucosal epithelium via the polymeric immunoglobulin receptor as secretory IgA antibodies (57–59); IgA is important in providing local protection against reinfection. Cohort studies of individuals infected with SARS-CoV-2 have shown that mucosal IgA is rapidly generated, continues to increase over time, is more durable in the saliva than in the blood, and can directly block virus infection (60–63). The presence of mucosal IgA correlates with a reduced risk of symptomatic infection, an effect that lasts more than 7 months (64, 65). Another study found that infection-induced mucosal IgA wanes after 9 months, and no substantial increase in mucosal IgA is induced after subsequent intramuscular vaccine boosting (65). The development of locally derived mucosal antibodies is important because circulating IgG (induced by infection or intramuscular vaccination) inefficiently penetrates the lumen of the respiratory tract (66, 67). Low levels of serum-derived anti-spike IgG in respiratory fluid in the absence of locally produced anti-spike IgA/IgM can provide a selective pressure that is readily overcome by mutational escape or a niche for replication that enables selection for enhanced transmission.

Plasmablasts are a specialized B cell subset derived from naive B cells or memory B cells that reencounter cognate antigen. These cells secrete high levels of antibodies into the circulation and tissues in response to SARS-CoV-2 infection or vaccination (68–72). Plasmablasts are generally short-lived cells, leading to a subsequent decline in circulating antibodies. In parallel, a population of plasma cells migrate to the bone marrow, where they constitutively secrete high levels of antibodies that gain access to the circulation (73–75). SARS-CoV-2 spike-specific plasma cells in the bone marrow are nonproliferating, with a subset representing a long-lived population of B cells (70). In peripheral lymphoid tissues, memory B cells are generated and can rapidly respond following reinfection or vaccine boosting. Several studies have shown that SARS-CoV-2 infection elicits a durable spike-specific IgG memory B cell pool that is detectable for longer than 6 to 8 months (39, 44, 76, 77), which may reflect persistent germinal center reactions in draining lymph nodes (78, 79). Altogether, SARS-CoV-2 infection induces humoral, mucosal, and memory B cell responses that can persist for extended time periods.

As mentioned above, the highest levels of protection appear to be achieved by a combination of infection- and vaccine-induced immunity, known as hybrid immunity (80). Globally, given the large numbers of infections experienced and vaccines administered, most individuals now have some form of hybrid immunity, although the heterogeneity in immunization/infection sequence and spike antigen exposure can result in different types of immune selective pressures. In one study, individuals with hybrid immunity were four to five times less likely to be infected during the period when Delta was dominant compared with those who had received two or three doses of mRNA vaccine (81). Individuals who received two or more doses of mRNA vaccine and then had an Omicron breakthrough infection exhibited higher levels of serum neutralizing antibody titers with greater breadth against heterologous Omicron subvariants compared with vaccination-only groups (82–84). Mechanistically, memory B cells, which are established following vaccination, are activated upon breakthrough infection, resulting in the preferential expansion of B cells that recognize conserved epitopes and the production of more broadly reactive and potently neutralizing antibodies (84–87). Nonetheless, de novo B cell responses are generated following Omicron variant booster vaccination or infection and can be further expanded by repeated exposures (88, 89).

### T Cell Immunity Against SARS-CoV-2

T cells also have crucial contributions to protection against recurring viral infections. CD4^+^ T cells differentiate into multiple T cell subsets, including T helper cells and T follicular helper cells. T follicular helper cells provide signals that enable B cells and their immunoglobulin genes to undergo somatic hypermutation to produce high-affinity and potently inhibitory antibody responses against a particular virus. CD8^+^ T cells differentiate into cytotoxic T lymphocytes and contribute to the clearance of infected cells by producing cytotoxic granules and antiviral cytokines.

Cohort studies of individuals infected with SARS-CoV-2 have shown that antigen-specific CD4^+^ and CD8^+^ T cells expand following infection and persist for months (44, 90–93). In recovered individuals, spike-specific CD4^+^ and CD8^+^ T cells in bronchoalveolar lavage (BAL) fluid samples are readily detected (67). The contribution of T cells to protective immunity has been highlighted by animal studies of SARS-CoV-2 infection. Both CD4^+^ and CD8^+^ T cells can be detected in the circulation, BAL fluid, and the lungs following the infection of rhesus macaques (66, 94–96). The use of cell-depleting antibodies showed CD8^+^ T cells to have a key role in controlling SARS-CoV-2 variant replication in vaccinated animals (97). Indeed, the more distant the challenge variant was from the immunizing antigen, the greater were the observed losses in serum neutralizing antibody activity, resulting in increased importance of T cell responses to protection (98). In mice, T cells also were important in controlling primary SARS-CoV-2 infection in the upper respiratory tract (e.g., nasal turbinates) and to a much lower extent in the lungs. Deep sequencing of persistent virus in the upper airway revealed the accumulation of mutations throughout the genome, suggesting that viral persistence within the upper respiratory tract can contribute to the evolution of variants (99).

Antigen-specific CD4^+^ T cells expand over the first few weeks after symptom onset followed by a slight decay, with an estimated half-life exceeding 200 days. SARS-CoV-2-specific CD4^+^ T cells, which broadly recognize peptides in the SARS-CoV-2 proteome, are primarily central memory T cells (CD4^+^CD45RA^−^CCR7^+^)and to a lesser extent effector memory T cells (CD4^+^CD45RA^−^CCR^−^)(39, 44). Antigen-specific CD4^+^ T cells show a Th1 bias with the expression of CXCR3^+^CCR6^−^ and are highly polyfunctional, as indicated by the concurrent detection of activation and effector molecules, including CD154, IFN-γ, IL-2, TNF-α, and, less frequently, granzyme B (44).

Antigen-specific CD8^+^ T cells expand during the first few weeks after symptom onset followed by a slight decay; their estimated half-life is nearly 200 days (44). During the early expansion phase, SARS-CoV-2-specific effector CD8^+^ T cells are polyfunctional; predominantly recognize the spike and nucleocapsid proteins; secrete IFN-γ, TNF-α, granzyme B, IL-2, or perforin; and persist for more than 6 months (44). A contraction phase of SARS-CoV-2-specific CD8^+^ effector memory T cells (CD8^+^CD45RA^−^CCR7^−^)is followed by or concurrent with an increase in T cell effector memory RA cells (CD8^+^CD45RA^+^CCR7^−^). Single-cell RNA sequencing of T cells from nasopharyngeal samples of SARS-CoV-2-infected patients shows increased frequencies of CD4^+^ and CD8^+^ T cells expressing IFN-γ, which correlate with reduced disease severity (100–102), although the antigen-specific status of these cells is not clear.

Hybrid immunity elicits a larger number of memory B and T cells than does vaccination alone (103), and immunization of previously infected individuals results in the expansion of T cell repertoires to include larger numbers of distinct clonotypes (104, 105); thus, hybrid immunity effectively increases the breadth of the cellular immune response. Hybrid immunity also generates immune responses directly in the respiratory mucosa, whereas most approved intramuscular vaccines confer systemic protection against severe disease but generate limited mucosal immunity (66, 67); the latter is required for restricting infection in the upper respiratory tract and preventing forward transmission. In studies using nonhuman primates, the induction of mucosal IgG, IgA, and T cell responses through mucosal boosting with adenoviral-vectored vaccines correlated with protection (106, 107). Similarly, in samples from human donors with hybrid immunity, BAL fluid contained elevated levels of spike-specific antibodies, memory B cells, and memory CD4^+^ and CD8^+^ T cells compared with samples from vaccination-only donors (66). Together, these data suggest that mucosal antigen exposure, especially in the context of hybrid immunity, leads to the most robust immune responses, leads to the greatest likelihood of protection against future SARS-CoV-2 variants, and, potentially, might prevent the evolution of new variants with greater transmissibility or escape potential.

## THE LANDSCAPE OF CORONAVIRUS EVOLUTION AND RELATIONSHIP TO IMMUNITY

**Figure 4:**
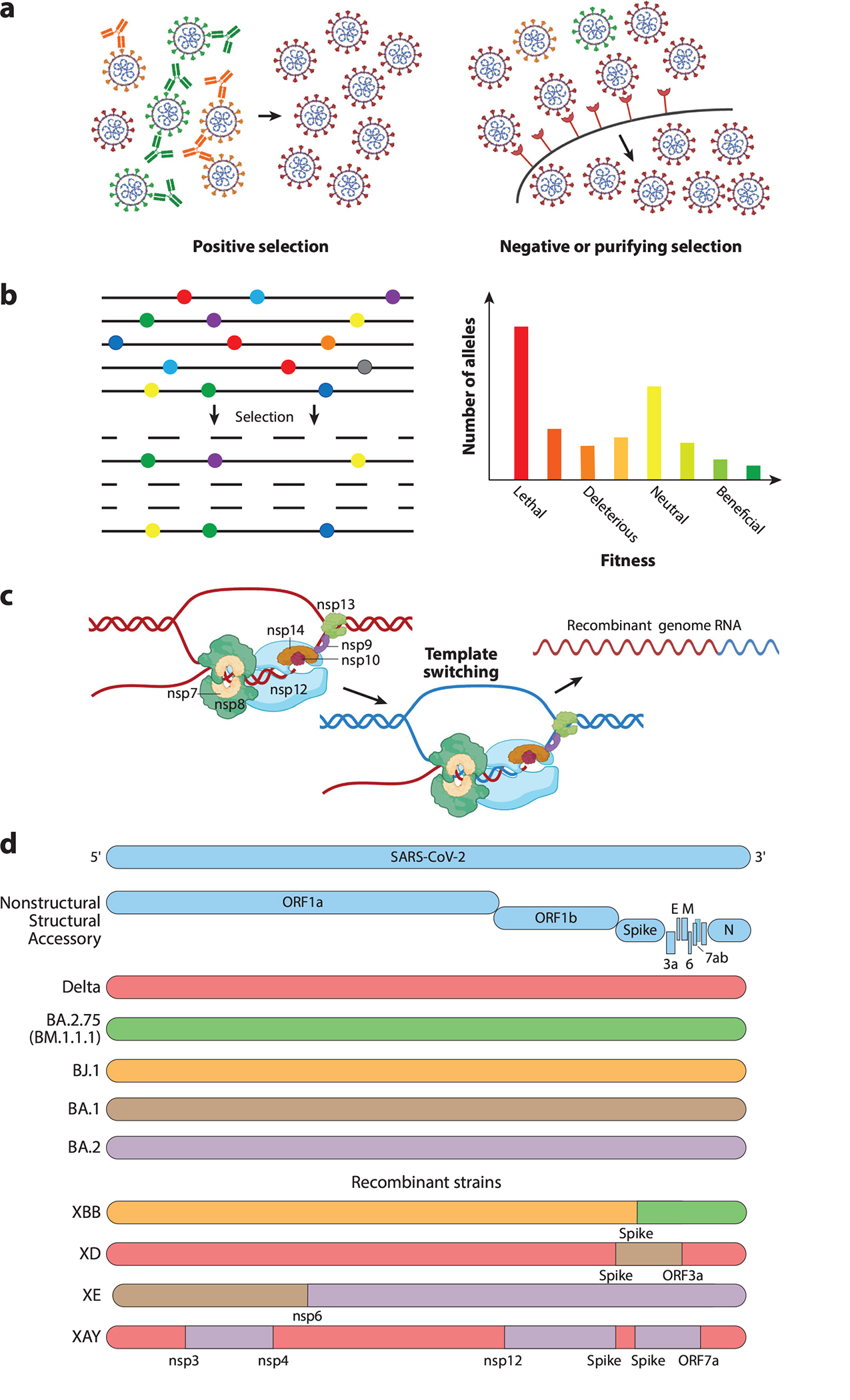
Mechanisms of SARS-CoV-2 evolution. (*a*) Within a virus population, antigenic drift results in the production of closely related variants that encode unique properties; these properties may be beneficial and increase in frequency (positive selection) or detrimental and decrease in frequency (negative or purifying selection) over time. (*b*) Evolutionary changes within a viral population are driven by fitness, which may lead to selection for strains encoding beneficial mutations and the removal of those encoding deleterious ones. (*c*) Coronaviruses encode a replicase composed of multiple proteins (e.g., nsps 7, 8, 9, 10, 12, 13, and 14), which together replicate and maintain the large RNA genome. Nonetheless, viral evolution, through point mutations, insertions, deletions, and/or recombination, creates genetic diversity. One potential recombination mechanism involves the dissociation of the replication complex from a partially replicated genome RNA and reassociation to the genome RNA of another strain present within the same cell. The result is a chimeric RNA genome, which may encode unique properties. (*d*) Notable SARS-CoV-2 recombination events that have occurred during the pandemic. The colored portion indicates the genome segments from each parental strain, and each breakpoint is denoted. Panels *a* and *c* adapted from images created with BioRender.com. Panel *d* based on the model in Reference 21.

CoVs encode the largest known genomes (27–32 kilobases) among RNA viruses. To maintain such a large RNA genome and not succumb to error catastrophe and deleterious mutations, CoVs have several proteins that help maintain the fidelity of the RNA replication process, including processivity cofactors (nsp7 and nsp8), an RNA helicase (nsp13), a unique proofreading enzyme for RNA virus replication (nsp14), and a nonenzymatic cofactor (nsp10). These proteins increase the overall replication fidelity of CoVs to levels approaching those of DNA-based organisms (8). While CoV RdRps, whose activity is encoded by nsp12, are intrinsically error prone and misincorporate nucleotides at rates similar to those of other RNA viruses, the 3′-to-5′ exoribonuclease activity encoded by nsp14 and cofactor nsp10 remove mismatched base pairs during RNA synthesis (108–110). By encoding a unique proofreading enzyme, CoVs have an overall coding capacity and a mutation rate that differ from those of other RNA viruses. Despite these fidelity mechanisms, seasonal CoVs gradually accumulate mutations through antigenic drift, which over time may shorten windows of protective immunity, resulting in repeated productive infections within target populations (111–113). Due to antigenic drift, variant strains with altered genome sequences may be selected based on their fitness advantage(s) for a given environmental pressure. In some instances, this selection may result in mutations that escape humoral immune responses, and, in others, adaptations that allow more efficient infection of host species may be selected (Figure 4a). This selection process can result in the emergence of more fit viruses, the purging of strains with deleterious mutations, and the emergence of new dominant variant strains (Figure 4b). Beyond antigenic drift, insertions and deletions, which are a product of replication errors and/or template switching between homologous RNAs, also can contribute to viral diversity, leading to more rapid viral evolution (114). In humans, the evolutionary rate of SARS-CoV-2 during the first two years of the COVID-19 pandemic was estimated to be between 2 × 10^−3^ and 4 × 10^−4^ substitutions/site/year (15, 20, 115–119), and rates were highest within the spike gene (0.81–1.08 × 10^−3^ substitutions/site/year), which was under the greatest selective pressures (120, 121). For comparison, the evolutionary rate of influenza A virus hemagglutinin and neuraminidase genes is estimated at 4.84 × 10^−3^ and 3.87 × 10^−3^ substitutions/site/year, respectively (122). Thus, the SARS-CoV-2 spike protein has evolved in humans at a rate that is fourfold to fivefold slower than that of influenza A virus surface proteins, which is likely due in part to the proofreading activity encoded within nsp14. Interestingly, exoribonuclease activity loss-of-function mutant viruses have not been experimentally recovered for MERS-CoV or SARS-CoV-2 despite attempts by multiple groups (123), suggesting an essential activity—an idea supported by a report showing that the SARS-CoV-2 nsp12 RdRp, in complex with cofactors nsp7 and nsp8 but without nsp14, exhibits low (10^−1^–10^−3^) intrinsic fidelity (124). These data contrast with those on the fidelity of RdRps for other RNA viruses, which is generally accepted to range between 10^−3^–10^−5^ (8, 125). One explanation for the inherently low fidelity rate of SARS-CoV-2 RdRp is its rate of RNA elongation (300–700 nt/s), which is the fastest known for a viral RdRp and rapid enough to replicate the entire ~30-kilobase genome in less than 2 minutes (126–128).

Recombination is an evolutionary tool used by many viruses and likely a contributor to cross-species adaptation of CoVs. While antigenic shift due to virus reassortment is a term typically used to describe swaps of genetic information in segmented RNA viruses such as influenza viruses, the nonsegmented CoV genome uses recombination to generate rapid changes in entire proteins/domains that otherwise would take a considerable amount of evolutionary time to evolve by point mutations and small insertions and deletions alone. For recombination to occur, two CoVs must coinfect the same cell at the same time. Given the number of CoVs that circulate in bats and other mammals, there is substantial potential for coinfection by related, yet distinct, CoV strains. However, the molecular mechanisms by which this process occurs are poorly understood. Recombination among human CoVs appears common, with estimates ranging from 0.06–0.3 recombination events per lineage per year (129). A role for nsp14 in this process was recently described for mouse hepatitis virus (130), a betacoronavirus in the *Embecovirus* subgenus that is distantly related to SARS-CoV-2 and other sarbecoviruses. During recombination, the RdRp, while bound to the nascent RNA strand, switches from one template strand to the corresponding sequence of another RNA strand, resulting in the production of a chimeric RNA molecule (Figure 4c). Also, due to the discontinuous subgenomic RNA replication strategy utilized by CoVs, nonhomologous recombination may occur during negative-strand RNA synthesis. Through these two recombination mechanisms, large portions of genetic information can be exchanged, potentially affecting species tropism, immune antagonism, and/or fitness. Whether the spillover of SARS-CoV-2 was facilitated by recombination is still unclear. Although multiple studies suggest that recombination between a pangolin CoV (Pang/GD) and a bat CoV (RaTG13) facilitated the emergence of SARS-CoV-2 (131, 132), others argue that recombination was not the primary evolutionary mechanism (133–135).

Recombination between variant strains occurs and represents a mechanism to acquire advantageous properties from different parental strains (21). Prior to the recombination of two BA.2-derived variants (BJ.1 and BM.1.1.1), which produced the XBB lineage (Figure 4d) (136, 137), multiple instances of intralineage recombination were detected but exhibited little impact on the trajectory of the pandemic (138). In contrast, upon the emergence of the XBB lineage in August 2022, XBB strains quickly became dominant. Relative to BA.2, XBB strains encoded an additional 14 mutations in the spike protein, including five in the N-terminal domain and nine in the RBD, which together confer extensive escape from vaccine-induced immunity and therapeutic monoclonal antibodies (mAbs) (136, 139). The XBB variant is a recombinant derived from the 5′ end of the BJ.1 strain and the 3′ end of the BM.1.1.1 strain, with a single breakpoint in the RBD (21). Through this recombination event, separate antibody escape mutations were inherited from BJ.1 (R346T, V445P, and G446S) and BM.1.1.1 (N460K, F486S, F490S, and an R493Q reversion) (Figure 2b) (21). While emerging variants rapidly replaced existing lineages during the early stages of the pandemic, currently, multiple genetically similar descendants of the major Omicron sublineages cocirculate. Whether they will continue to gradually drift or whether additional recombination events will occur, resulting in unique constellations of spike mutations, remains to be seen.

### Selective Pressures Driving SARS-CoV-2 Evolution

Mutations in the SARS-CoV-2 spike protein that affect hACE2 binding affinity and adaptive immune evasion explain the vast majority of SARS-CoV-2 evolution in strains that infect humans (140). While other factors can contribute to SARS-CoV-2 evolution across the genome, including replication efficiency, duration of infectiousness, innate immune evasion, and tissue tropism (141), in this section, we limit our discussion to the two primary drivers of spike protein adaptation, which are intrinsic transmissibility and adaptive immune evasion.

### Transmissibility

Virus strains transmit between hosts with different efficiencies, which is measured by their basic reproductive number (R_0_) (12). R_0_ is an epidemiologic metric used to describe the contagiousness or transmissibility of infectious agents (142). Between 2020 and 2022, at least six distinct SARS-CoV-2 variants emerged with successively higher R_0_ values (D614G, Alpha, Beta, Gamma, Delta, and Omicron) (143–145). Analysis of SARS-CoV-2 genomes during this period suggests that the primary drivers of SARS-CoV-2 evolution and fitness were hACE2 receptor affinity and humoral immune evasion (140). In the first months of the pandemic, primary selection was for adaptations that maximized transmission (146, 147). The D614G substitution in the spike protein, which has remained fixed in all subsequent dominant variants, enhances transmission and infectivity in humans by increasing hACE2-binding affinity (13, 148–151). Beyond D614G, an N501Y mutation located within the RBD is the only other shared spike mutation in the Alpha, Beta, and Gamma variant strains, and it enhances viral infection of human airway epithelial cells and transmission in a hamster model of SARS-CoV-2 (152). The Delta variant, which has higher replication efficiency in primary human airway epithelial cultures (153, 154), is estimated to have been 60% more infectious in humans compared with prior variants and resulted in a global surge in COVID-19-related hospitalizations in 2021 (17, 155–157). Increases in transmissibility for the Delta variant are at least partially due to higher spike protein affinity for hACE2 than is observed for the D614G strain; this affinity is mediated by a T478K mutation that stabilizes a loop in the receptor-binding motif (S473–490) (Figure 2b) (158, 159). For the initial Omicron variant lineages, the R_0_ was roughly 2.5-fold higher than that of Delta (160); multiple mutations that confer greater hACE2 affinity emerged in these strains. BA.1.1, which is derived from the BA.1 lineage, demonstrates the highest degree of hACE2 binding among the initial Omicron variants, followed by BA.2 = BA.5 > BA.3 = BA.1 (161, 162). Structural and biochemical analyses revealed that the enhanced receptor affinity for BA.1.1 was conferred by an R346K mutation, which increases the number of salt bridges and long-distance interactions between the RBD and hACE2. Alternatively, the absence of a G496S mutation in BA.2 and BA.5 prevents disruption of the local RBD-hACE2 interaction network, resulting in higher affinity despite the absence of the R346K mutation (162). Omicron subvariants such as BA.2.75, BQ.1.1, XBB.1.5, EG.5.1, HK.3, and BA.2.86, but not JN.1, have single-digit nanomolar affinities that are enhanced relative to parental BA.2 and BA.5 strains (137, 163, 164).

Mutations that alter proteolytic cleavage of the spike protein occur in a subset of variants and relate to transmissibility. As SARS-CoV-2 progeny are packaged through the endoplasmic reticulum–Golgi intermediate compartment in infected cells, spike proteins are cleaved proteolytically by the host protease furin at a polybasic amino acid sequence known as the furin cleavage site (FCS) (6). The result is the release of fully infectious virions capable of entering cells by receptor-mediated endocytosis or direct membrane fusion, which, depending on the route of viral entry, can affect SARS-CoV-2 infectivity, pathogenesis, and transmission (165, 166). Initial SARS-CoV-2 strains encoded an FCS sequence that was not efficiently processed by human furin. Over the course of the pandemic, the FCS, which is notably absent in other sarbecoviruses, evolved. For example, in the Alpha and Delta variants, which encode P681H and P681R substitutions (Figure 2b), respectively, the spike protein is cleaved with almost 100% efficiency and contributes to enhanced transmissibility (167, 168). Omicron variants and their descendant lineages also encode P681H or P681R mutations, which, in conjunction with other mutations, skew the principal method of viral entry from transmembrane protease serine 2–mediated direct membrane fusion to cathepsin-mediated endosomal entry (169).

SARS-CoV-2 is primarily transmitted from infected individuals via small respiratory droplets and aerosols (170). For influenza viruses, aerosol transmission is a consequence of replication in cells of the upper respiratory tract (171), which is a notable characteristic of Omicron subvariants (172). In ex vivo cultures, BA.1 and BA.2 variants replicate more efficiently in human bronchi and nasal tissues but less well in lung tissues compared with Delta strains (172–174). Moreover, Omicron-infected individuals exhibit increased aerosol viral shedding compared with those infected with pre-Omicron variants, independent of vaccination or prior infection status (175). Thus, adaptation to anatomic sites more amenable to respiratory transmission may have provided initial Omicron lineages with a competitive advantage over Delta.

### Effects of Viral Evolution on Immunity

In the first few years of the pandemic, the primary driver of initial SARS-CoV-2 variant evolution was transmissibility, most likely due to the large population of SARS-CoV-2-naive individuals. As many humans became antigen experienced through vaccination or infection, an increasing number of changes were selected to escape humoral immune pressures.

#### Humoral immunity.

RNA viruses commonly exhibit substantial antigenic variation, which provides a mechanism for escaping humoral immunity, thereby effectively reestablishing susceptible populations (112, 176, 177). First-generation SARS-CoV-2 vaccines utilizing the Wuhan-1 spike sequence such as mRNA-1273 were 94% and 100% effective against infection and severe disease (178), respectively. Following the emergence of SARS-CoV-2 variants Alpha through Delta, efficacy of mRNA (mRNA-1273 and BNT162b2) and adenoviral-vectored (ChAdOx1 and AZD1222) first-generation SARS-CoV-2 vaccines remained high (70–91% and 94–96%, respectively) against symptomatic infection and severe disease (179, 180). Serum neutralizing antibodies induced by natural infection or vaccination correlate with protection against SARS-CoV-2 and are a strong selective pressure on viral evolution (181, 182). Thus, the efficacy of these vaccines against early variants was maintained likely due to the relatively small reductions in vaccine-elicited neutralizing antibodies (183).

The first evidence of antigenic evolution for SARS-CoV-2 was the emergence of variants encoding K417T/N and E484K (Beta and Gamma), N501Y (Alpha, Beta, and Gamma), or L452R (Delta) mutations, which escaped therapeutic mAbs and impaired the neutralizing activity of polyclonal sera derived from vaccinated or infected individuals (Figure 2b) (184–190). SARS-CoV-2-specific antibodies are commonly binned into one of four classes: Class 1 blocks ACE2 binding and is accessible in RBD “up” conformation, Class 2 blocks ACE2 binding and is accessible in RBD “up” or “down” conformations, Class 3 does not block ACE2 binding and is accessible in RBD “up” or “down” conformations, and Class 4 does not block ACE2 binding and is accessible in RBD “up” conformation (191). While K417T/N mutations escape some Class 1 antibodies, the effects on polyclonal serum antibody binding and neutralization were modest (185, 186, 192–194). Residue E484 within the SARS-CoV-2 RBD is immunodominant, and mutation to K, P, or Q amino acids results in markedly decreased neutralizing activity by many inhibitory Class 2 antibodies (186). In comparison, the L452R mutation is associated with reductions in neutralization potency for Class 3 mAbs and vaccine-elicited sera; however, this mutation also increases spike affinity for ACE2 through indirect electrostatic interactions (186, 190, 195).

Concerns of viral escape from passively administered mAbs or vaccine-elicited antibodies were realized with the emergence of the highly mutated initial Omicron lineages (BA.1–BA.5) (196–200), which displayed a greater capacity for breakthrough infections in fully vaccinated individuals (201–205). Protection against symptomatic infection by BA.1 was 65.5% at 2 to 4 weeks after two doses of BNT162b2 but dropped to 8.8% at 25 or more weeks (206). Similarly, mRNA-1273 was only 44% effective against BA.1 infection at 4 weeks but decreased to 5.9% at 7 months after the second dose (207). The loss in vaccine efficacy corresponded to substantive (14- to 35-fold) reductions in serum neutralizing titers (208), which also were seen following a two-dose primary series with NVX-CoV2373 or AZD1222 (209). Similar to previous variants, BA.1, BA.2, and BA.3 encoded substitutions K417N, E484A, and N501Y, which primarily mediated neutralizing antibody escape, but also included other substitutions, such as G339D, N440K, S477N, T478K, and Q498R (210). Acquisition of L452R and F486V and reversion of R493Q in BA.5 led to further compromise of mAb- and vaccine-elicited sera neutralization (22, 202–205, 210–212). L452R contributed to escape from Class 2 and some Class 3 mAbs, whereas F486V promoted escape from Class 1 and 2 mAbs but also reduced ACE2 affinity, which was restored by the compensatory R493Q reversion (213). For mRNA vaccines, efficacy against several initial Omicron sublineage variants, including BA.1, BA.2, BA.2.12.1, BA.3, and BA.4/5, was partially restored by a booster dose, which temporarily increased neutralizing antibody titers (209, 214–216).

Since 2022, subvariants descending from BA.2 have exhibited convergent evolution to escape from parental vaccines. Initially, to combat this, bivalent boosters incorporating Omicron variants were developed for the mRNA and recombinant protein subunit vaccines. However, effectiveness against symptomatic infection by subsequent Omicron strains was relatively low. In one study of people who received a Wuhan-1/BA.5 bivalent mRNA booster dose, the overall vaccine efficacy was 34% against symptomatic infection, independent of the number of prior doses, days since the last dose, or age of the recipient (217). Individuals receiving a bivalent mRNA booster exhibited lower (13- to 155-fold) serum neutralizing titers against BA.2.75, BQ, and XBB subvariants than against historical SARS-CoV-2 strains (137, 218). One possible explanation for these results is that because bivalent boosters included the ancestral Wuhan-1 spike, responses may have been biased toward imprinted memory B cells rather than the expansion of de novo type-specific B cells (22, 23, 136, 210, 212, 218–220). More recently, BA.2.86 and JN.1 variants have emerged with additional spike antibody evasion mutations at residues K356, R403, N450, Y453, L455, F456, and N481, along with a reversion of A484K, which utilizes an escape substitution present in earlier variants (221, 222). Although the mechanisms that dictate B cell differentiation upon antigen recall are still actively being investigated, recent evidence suggests that upon antigen reexposure, the titer and affinity of circulating antibodies, as well as the amount of antigen, affect the recruitment of naive B cells and generation of type-specific responses (223, 224). Nonetheless, repeated breakthrough Omicron infections can overcome imprinting and result in type-specific antibody responses against Omicron viruses (88, 89, 225, 226), suggesting that vaccines should be updated periodically to include the prevailing variants, which could expand individual B cell repertoires to include antibodies against the escape regions noted above. Given these findings and the heterogeneity of preexisting immunity that now exists, future vaccination strategies may also need to incorporate epitope masking and/or antigenic variation strategies to overcome immune imprinting and induce optimal protection against contemporary variants.

XBB.1.5 spike-expressing booster formulations, including the Pfizer and Moderna mRNA and Novavax protein subunit vaccines, are currently administered in the United States and elsewhere, and it is anticipated that even newer Omicron variant–targeted vaccines may be mandated for late 2024 and beyond. While vaccine efficacy data are limited, initial reports suggest that the XBB.1.5-based monovalent booster is more effective than the previous Wuhan-1/BA.5 bivalent boosters (227, 228). In studies performed in the United States and the Netherlands between September 2023 and January 2024, vaccine effectiveness against symptomatic infection was approximately 10% greater with the XBB.1.5-based monovalent booster than that observed with the bivalent booster in 2022 (229, 230). The monovalent XBB.1.5 booster is likely a better match against currently circulating strains; vaccination of previously uninfected individuals resulted in increased serum neutralizing titers against XBB subvariants, including XBB.1.5 (27-fold), EG.5.1 (28-fold), and JN.1 (13-fold) (231).

#### Cellular immunity.

Considering that T cell immunity also controls SARS-CoV-2 infection (232, 233), there is the potential for selective pressure to escape these responses. In contrast to humoral immune responses against SARS-CoV-2, which is primarily directed against the RBD, cell-mediated immunity is directed against many targets across the viral proteome, including the spike, membrane, nucleocapsid, nsp3, nsp4, ORF3a, and ORF8 proteins (91). While the potential impact of antigenic diversity on neutralizing antibody function is significant, there is less of an effect on T cell–mediated immunity, at least at the population level, which is not surprising given the number of recognized peptides and the heterogeneity of MHC allele expression (19). Nonetheless, some mutations that escape T cell responses elicited by natural infection and/or vaccination have been described: (*a*) CD8^+^ T cell escape mutations: S:P272L, which escapes the most frequent Class I HLA allele globally, HLA A*02 (234); S:L452R and S:Y453R, which both escape Class I HLA-A*24:02 (195); ORF3a:Q213K, which escapes Class I HLA-A*01:01; and N:P13L/S/T, T362I, and P365S, which escape Class I HLA-B*27:05, -A*03:01, and -A*11:01, respectively (235); and (*b*) CD4^+^ T cell escape mutations: S:D80A, D215G, and D1118H, which escape Class II HLA-DRB1*1501, -DRB1*0301, -DRB1*0401, -DRB1*1301, and -DRB1*1501 (236, 237).

Despite such instances of escape, bioinformatic analyses incorporating experimentally defined and validated peptides that were referenced against more than 13 million SARS-CoV-2 genomes suggest that CD4^+^ and CD8^+^ T cell epitopes are not under strong diversifying selection (19). These findings are supported by studies on human samples from convalescent and vaccinated individuals, which show that 93% and 97%, respectively, of the CD4^+^ and CD8^+^ T cell epitopes elicited are conserved in the Alpha, Beta, and Gamma variants (238). Also, despite the relatively large number of mutations encoded by Omicron variants, there is no correlation in mutation frequency between dominant or subdominant T cell epitopes (239), and T cell responses elicited by multiple vaccine platforms are preserved in more than 80% of individuals (240, 241). While the number of reports on T cell responses directed against more recently circulating Omicron variants is limited, breakthrough infection by early Omicron subvariants in BNT162b2 mRNA-vaccinated individuals results in CD8^+^ T cell responses that still can respond to descendent lineages such as XBB (242). Despite demonstrating a high capacity to escape vaccine-elicited polyclonal neutralizing responses, one dose of bivalent mRNA booster retained 58–70% efficacy against hospitalization (243, 244). Consistent with these data, ex vivo stimulation of T cells isolated from bivalent mRNA–boosted recipients responded to both ancestral and XBB spike proteins (245). HLA binding analyses between the ancestral and XBB.1.5 spike sequences suggest that differences in BA.2.86 and JN.1 spike sequences are either absent or limited in experimentally defined T cell epitopes (246). Together, these studies suggest that cellular immunity—or, additionally, immunity conferred by non-neutralizing Fc effector function antibodies directed against Omicron sublineage variants—is maintained in the context of low or waning neutralizing antibody levels. The available data suggest that SARS-CoV-2 evolution is not principally driven by T cell escape, resulting in largely preserved cell-mediated immunity across variants. Given the diversity of HLA alleles in humans, the degree of viral diversity needed for T cell escape across the population may exceed the limits required to maintain viral fitness (233).

Considering the complexity of the current immune landscape, which is the product of natural infections with a spectrum of SARS-CoV-2 variants and historical and updated vaccines, the prospect of continued antigen drift versus shift remains an open question. Despite unique proofreading mechanisms to limit the number of deleterious mutations that occur during genome replication, SARS-CoV-2 retains the capacity to rapidly adapt to new host species and immune settings. Current vaccination approaches, which elicit systemic immunity through intramuscular administration, may allow infection and transmission cycles to continue due to the relatively low levels of mucosal immunity that are induced (67). As a result, SARS-CoV-2 evolution, and the emergence of variants with increased fitness, will likely continue until therapeutic or vaccine-induced sterilizing immunity can be achieved.

## FUTURE PERSPECTIVES

The COVID-19 pandemic has expanded our understanding of viral evolution and the immune and host selective pressures that drive the emergence of viral variants. In the future, advances in artificial intelligence and structural biology may help predict variants and their relative fitness in real time (247, 248). Viral evolution may be most easily conceptualized as the response to the pressures that results in the greatest level of fitness for a given set of conditions. Hence, whether a given variant is more or less fit than another is relative and depends on the environment and collective immune conditions. For example, had they cocirculated in late 2020, would the initial Omicron variant lineage have outcompeted Alpha to become the dominant strain globally? Given that most of the world was antigenically naive at the time, the mutations conferring immune evasion that are present in Omicron may not have overcome the high intrinsic transmissibility of Alpha. Indeed, in naive mice, BA.1 was outcompeted by the Alpha and Delta variants. Yet, BA.1 competition was restored when mice were vaccinated prior to challenge (249). Therefore, correctly predicting variant fitness will require careful attention to ensure that all variables are accurately considered.

At present, four SARS-CoV-2 mucosal vaccines have been deployed in various parts of the world, although efficacy data for each are limited (250). Nonetheless, in animal models and humans, mucosal vaccination confers greater control of infection in respiratory tissues, and protection correlates with mucosal and systemic IgA, IgG, and T cell immunity (106, 251). Therefore, an important objective going forward will be to reproduce the benefits associated with hybrid immunity using a combination of systemic and mucosal vaccination strategies.
